# Studies of recombinant TWA1 reveal constitutive dimerization

**DOI:** 10.1042/BSR20160401

**Published:** 2017-01-13

**Authors:** Ore Francis, Genevieve E. Baker, Paul R. Race, Josephine C. Adams

**Affiliations:** 1 School of Biochemistry, University of Bristol, Bristol BS8 1TD, U.K.; 2 BrisSynBio Synthetic Biology Research Centre, University of Bristol, Bristol BS8 1TQ, U.K.

**Keywords:** protein purification, TWA1, LisH, CTLH, Ubiquitin

## Abstract

The mammalian muskelin/RanBP9/C-terminal to LisH (CTLH) complex and the *Saccharomyces cerevisiae* glucose-induced degradation (GID) complex are large, multi-protein complexes that each contain a RING E3 ubiquitin ligase. The yeast GID complex acts to degrade a key enzyme of gluconeogenesis, fructose 1,6-bisphosphatase, under conditions of abundant fermentable carbon sources. However, the assembly and functions of the mammalian complex remain poorly understood. A striking feature of these complexes is the presence of multiple proteins that contain contiguous lissencephaly-1 homology (LisH), CTLH and C-terminal CT11-RanBP9 (CRA) domains. TWA1/Gid8, the smallest constituent protein of these complexes, consists only of LisH, CTLH and CRA domains and is highly conserved in eukaryotes. Towards better knowledge of the role of TWA1 in these multi-protein complexes, we established a method for bacterial expression and purification of mouse TWA1 that yields tag-free, recombinant TWA1 in quantities suitable for biophysical and biochemical studies. CD spectroscopy of recombinant TWA1 indicated a predominantly α-helical protein. Gel filtration chromatography, size-exclusion chromatography (SEC) with multi-angle light scattering (SEC-MALS) and native PAGE demonstrated a propensity of untagged TWA1 to form stable dimers and, to a lesser extent, higher order oligomers. TWA1 has a single cysteine residue, Cys^139^, yet the dimeric form was preserved when TWA1 was purified in the presence of the reducing agent tris(2-carboxyethyl)phosphine (TCEP). These findings have implications for understanding the molecular role of TWA1 in the yeast GID complex and related multi-protein E3 ubiquitin ligases identified in other eukaryotes.

## Introduction

The muskelin/RanBP9/CTLH complex (referred to subsequently as the MRCTLH complex) is a large multi-protein complex that has been identified in cells from several mammalian species [1,2]. The protein components of this complex include muskelin, RanBP9/RanBPM, TWA1, MAEA, Rmnd5a and Armc8 [1]. Molecular cell biological and gene knockout or knockdown studies of individual proteins indicate a wide repertoire of activities that include roles of muskelin in cell morphology, transcriptional regulation and synaptic processes [3–5]; of RanBP9 in ERK signalling, neurodegeneration and spermatogenesis [6–8]; and of Armc8 in modulating stability of hepatocyte growth factor-regulated tyrosine kinase substrate or β-catenin [9,10]. *Xenopus tropicalis* Rmnd5 has been demonstrated to have E3 ubiquitin ligase activity *in vitro* and to be important for embryonic development of the *Xenopus* mesencephalon, prosencephalon and eye *in vivo* [11]. However, how the specific role(s) of each protein relate to the assembly and function of the complex remains unclear.

A homologous complex in the budding yeast *Saccharomyces cerevisiae* is termed the glucose-induced-degradation deficient (GID) complex and has been studied in detail. The 600 kDa GID complex is a multi-protein E3 ubiquitin ligase that is activated by the association of an additional protein, Gid4, to poly-ubiquitinate gluconeogenic enzymes such as fructose 1,6-bisphosphatase and targets them for proteosomal degradation following a shift of yeast cells from a non-fermentable carbon source to a fermentable carbon source [12]. The constitutive GID complex includes Gid1, a large multi-domain scaffolding protein (orthologous to RanBPM/RanBP9); an enzymatically active RING E3 ligase Gid2 (orthologous to Rmnd5a); an enzymatically inactive variant RING protein Gid9 (orthologous to MAEA), which dimerizes with Gid2 and is necessary for Gid2 activity; Gid5 (orthologous to Armc8); Gid7, which is bound by Gid4 to activate the complex and a small scaffolding protein Gid8, (orthologous to TWA1) that interacts with several other proteins in the complex [13]. Gid1, Gid4 and Gid5 also function in vacuole-mediated degradation of fructose 1,6-bisphosphatase, a pathway equivalent to metazoan endo-lysosomal protein degradation [14]. RanBPM-binding proteins have been identified in the plant *Arabidopsis thaliana*, which include orthologues of TWA1/Gid8, MAEA/Gid9, Rmnd5/Gid2 as well as an orthologue of mammalian WDR26, which has domains in common with muskelin and Gid7 [15]. The Rmnd5/Gid2 orthologue of the plant *Lotus japonicus* has E3 ligase activity *in vitro* [16]. Collectively, these observations implicate E3 ligase activity as a conserved property of these distinct yet related multi-protein complexes identified in different lineages of eukaryotes.

**Figure 1:**
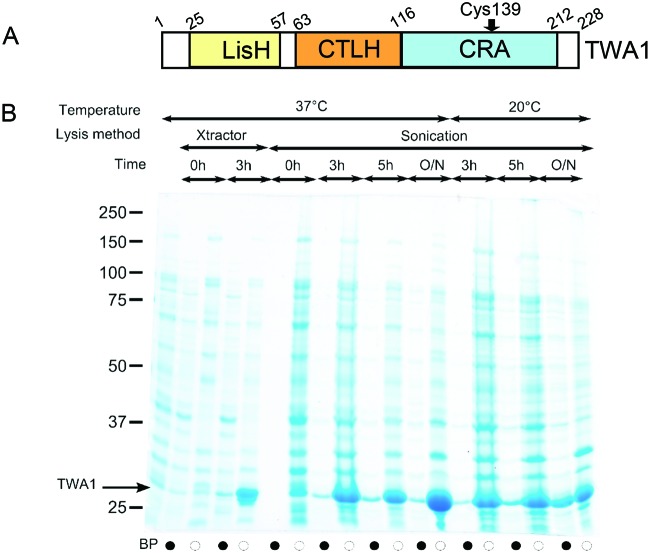
TWA1 and set up of bacterial expression **(A)** Schematic diagram of the domain organization of TWA1. Numbers are based on the human and mouse proteins, which have 99% sequence identity. (**B**) SDS/PAGE analysis of lysate supernatants (black circles) or lysate pellets (white circles) from TWA1.V5His6-expressing bacteria grown at the indicated temperatures, without or with induction with 1 mM IPTG; BP = bacterial pellet of uninduced cells. Lysates were prepared with commercial buffer (Xtractor) or by sonication. Samples were analysed under reducing conditions on SDS/PAGE (12% gel) and the proteins visualized by Coomassie blue staining.

We characterized previously *in silico* that many of the protein components of the GID and MRCTLH complexes are present in multiple lineages of extant eukaryotes, indicative of an origin in the last eukaryotic common ancestor [17]. Another striking feature of the MRCTLH, GID and *Arabidopsis* complexes is the related domain composition of many of the constituent proteins. RanBP9/Gid1, Rmnd5/Gid2, TWA1/Gid8 and MAEA/Gid9 are all multi-domain proteins that include, in the same domain order, a lissencephaly-1 homology (LisH) domain, a C-terminal to LisH (CTLH) domain and a C-terminal CT11-RanBP9 (CRA) domain. The LisH and CTLH domains are both characterized as largely α-helical regions. A conserved feature of LisH is the presence of N-terminal and C-terminal α-helices connected by an apical loop [17]. In lissencephaly-1 (Lis1), LisH mediates assembly of anti-parallel dimers [18]. The CTLH domain was identified as an α-helical region frequently found C-terminal to a LisH domain [19]. CTLH domains are predicted to contain three α-helices, separated by short loops [17]. The CRA domain is a region of approximately 100 amino acids that includes six α-helices [17,20]. These three domains comprise the entirety of TWA1/Gid8 (Figure 1A), whereas RanBP9, Rmnd5 or MAEA each contain additional domains [13,17]. Muskelin, which is not encoded in *S. cerevisiae*, is a RanBP9-binding protein in the MRCTLH complex and also contains LisH and CTLH domains, as do Gid7 and WDR26 [3,12,15]. These shared attributes of domain architecture suggest that LisH and CTLH domains could be important for the assembly and function of the complex. In general, LisH domains participate in protein oligomerization by the formation of anti-parallel dimers [21–26]. However, there are also indications that the LisH domains of MRCTLH proteins can have distinct functions. The LisH domain of muskelin includes a cryptic nuclear localization sequence that is likely to be activated by phosphorylation of a conserved threonine residue adjacent to the C-terminus of muskelin [3]. Sequence analyses of the LisH and CTLH domains of MRCTLH proteins have indicated that the LisH and CTLH domains of TWA1 and RanBP9 have related conserved features, whereas these domains in MAEA and Rmnd5 have different profiles of conserved residues [17].

For further investigation of the structural/functional relationships of the LisH, CTLH and CRA domain organization, TWA1/Gid8 is of particular interest because it is the smallest protein within the complex that has LisH, CTLH and CRA domains (Figure 1A), because of its presence in all forms of this ubiquitin E3 ligase complex identified to date and because of its very high conservation across eukaryotes [1,2,12,15,17]. However, relatively little is known about the functional roles of either TWA1 or Gid8. TWA1 is a direct binding partner of RanBP9 [2] and is present in both the nucleus and cytoplasm of mammalian cells [17]. Similarly, Gid8 interacts directly with Gid1 [13]; in addition, *gid8* deletion mutants in *S. cerevisiae* indicated that Gid8 functions as an adapter for Gid2 and Gid9 [13]. An *in silico* prediction of protein–protein domain interactions in *S. cerevisiae* implicated a strong involvement of Gid8 within a “core” GID complex: tandem affinity purification of Gid1 and the loss of several Gid1-co-purifying proteins in a *Gid8* gene deletion yeast strain (ΔYMR135C) give substance to this hypothesis [27]. Whereas LisH domains from several unrelated proteins have been crystallized [18,24,28] and the LisH domain of muskelin has been crystallized in combination with its N-terminal discoidin domain [29], almost nothing is known about the biochemical properties of TWA1, Gid8 or their orthologues in other eukaryotes. In transient expression experiments in mammalian COS-7 cells, we noted that TWA1 could be expressed to higher levels than the RING domain-containing proteins, Rmnd5 or MAEA [17]. Given the potential importance of LisH, CTLH and CRA domains for the activities of multiple proteins within the MRCTLH or GID complexes, we established a system for bacterial expression and purification of TWA1 and report here on a biochemical study of the purified TWA1 protein.

## Materials and methods

### Molecular biology procedures

TWA1 cDNA (from mouse, 99% amino acid sequence identity to human TWA1) was amplified by PCR from a previously described plasmid [17], with DNA oligonucleotide primers 703F (5′-CACCATGAGTTATGCAGAAAAACCCG-3′) and 268R (5′-GATCGAATTCGCTCTACTTGGGCTCCTC-3′) (Sigma–Aldrich, U.K.) and ligated into pET151/D-TOPO bacterial expression plasmid (Invitrogen) following the manufacturer’s instructions. This plasmid supplies an N-terminal V5His6 tag with a following Tobacco Etch Virus nuclear inclusion α endopeptidase protease (TEV) cleavage site to the encoded protein.

### Pilot experiments for TWA1 expression in *Escherichia coli*

Unless otherwise stated, chemicals were from Sigma–Aldrich. IPTG induction of recombinant TWA1.V5His6 expression in *E. coli* BL21(DE3) cells (Invitrogen) was performed on 5 ml cultures to elucidate appropriate conditions for TWA1.V5His6 expression. Single *E. coli* colonies were inoculated into LB containing 100 µg/ml ampicillin (LB/amp) and grown overnight at 37°C with shaking. The next day, 1 ml of each culture was used to inoculate 9 ml of fresh LB/amp medium. Cultures were incubated at 37°C until an *A*_600_ between 0.4 and 0.6 was reached. Then, 500 μl of each culture was collected, centrifuged at 13000 rpm for 2 min and the pellet was stored at −20°C as the ‘0h’ sample. The remaining culture was split into two equal portions. Expression of TWA1.V5His6 was induced in one of these by the addition of IPTG to a final concentration of 1 mM. Growth was continued at 37°C and 1 ml of each culture was collected at designated times up to 16 h (overnight). At each timepoint, the bacteria were pelleted by centrifugation and stored at −20°C. In a parallel experiment, the cultures were grown at 20°C after IPTG induction. To compare the extractability of TWA1.V5His6 using different methods of bacterial cell lysis, bacterial pellets corresponding to 0 h or 3 h timepoints were either lysed by gentle agitation in 500 μl of Xtractor buffer (Clontech) at room temperature for 1 h or resuspended in 1 ml of lysis buffer (200 mM NaCl, 50 mM TRIS, pH 8.0, 10% glycerol, 25 mM imidazole and 2 mM PMSF) and disrupted by sonication for 2 min (four cycles of 5 s bursts at 25 s intervals) on ice. All lysed samples were centrifuged at 18000 ***g*** for 30 min at 4°C to separate cell supernatants from residual pellets. Pellets were resuspended in 100 μl 2× SDS/PAGE sample buffer containing 100 mM DTT and supernatants were mixed 1:1 with the same buffer. Thirty microlitres of each sample were resolved on SDS/PAGE (10% gel) under reducing conditions. Proteins were visualized on gels by Coomassie blue-based staining with Gelcode® Blue (Thermo Fisher).

### Expression and purification of TWA1.V5His6

For expression of TWA1.V5His6 at larger scale, a single colony from LB–ampicillin plates streaked with *E. coli* BL21(DE3) cells transformed with pET151-/D-TOPO:TWA1.V5His6 was added to 10 ml LB/amp medium and the culture grown overnight at 37°C. This culture was then transferred into 1 litre of fresh LB/amp medium and grown at 37°C until an *A*_600_ of 0.6 was reached. IPTG was added to a final concentration of 1 mM, and cultures were grown for 5 h at 18–20°C, harvested by centrifugation (10000 ***g*** for 30 min at 4°C) and resuspended in 40 ml of cold lysis buffer consisting of 200 mM NaCl, 50 mM TRIS, pH 8.0, 10% glycerol, containing 25 mM imidazole and 2 mM PMSF. Cells were disrupted by sonication for 8 min (eight cycles of 15 s bursts at 45 s intervals) on ice and centrifuged at 18000 ***g*** for 30 min at 4°C. The cell supernatant was applied to a His-trap FF column (GE Healthcare), after stripping with EDTA and reloading with NiSO_4_, connected to an Akta System (GE Healthcare) and allowed to flow through, resulting in binding of TWA1.V5His6 to the column. After binding, lysis buffer and elution buffer (200 mM NaCl, 50 mM TRIS, pH 8.0, 10% glycerol, containing 1 M imidazole and 2 mM PMSF) were applied to the column to establish a 25 mM to 1 M imidazole gradient. Protein elution was monitored by protein UV absorbance detection at 280 nm, and the eluent was collected as 1 ml fractions. The purity of the protein preparation in each fraction was assessed by SDS/PAGE using NuSEP TRIS/glycine gels (Generon). Fractions containing TWA1.V5His6 were pooled and dialyzed against 50 mM EDTA, 1 mM TRIS overnight. The following day, 25 µg of TEV protease (recombinant protein, the gift from Skye Hodson and Steven Burston, University of Bristol) was added to the pooled fractions to cleave off the N-terminal tags. This was achieved by dialysing the mixture against fresh TEV buffer overnight. The mixture was then loaded on a 5 ml HisTrap FF column to bind the His6-tagged protease, yielding cleaved, tag-free TWA1 in the flow through. Flow-through fractions were assessed for protein purity on NuSEP TRIS–glycine gels. The peak TWA1 containing fractions were pooled and concentrated to 2–3 ml by ultrafiltration through a 10 kDa cut-off membrane (Amicon Ultra-15 Millipore). TWA1 was further purified by gel filtration on a HiLoad 16/600 Superdex 75 pg column, with column bed volume of 120 ml (GE Healthcare), in a buffer of 200 mM NaCl, 50 mM TRIS, pH 8.0. Eluted fractions were assessed for purity by SDS/PAGE. The TWA1-positive fractions were pooled and concentrated by ultrafiltration through a 10 kDa pore membrane to a concentration of 10 mg/ml and stored at 4°C for subsequent experiments. Molecular weight standards applied to the same column included alcohol dehydrogenase (80 kDa), HLA-A2/β2-microglobulin (46 kDa), CD8a (30 kDa) and β2-microglobulin (11 kDa). Protein concentrations were measured with a NanoDrop Lite spectrophotometer (Labtech).

### Circular dichroism

CD spectroscopy measurements were conducted using a JASCO J-810 spectropolarimeter fitted with a Peltier temperature controller (Jasco, U.K.). Purified recombinant TWA1 was prepared at 5 µM in PBS. Full CD spectra were recorded from 240 nm to 190 nm in 0.1 mm path-length quartz cuvettes at 5°C. The instrument was set with a scan rate of 100 nm/min, a 1 nm bandwidth and a 1 s integration time. Scans were retrieved and data analysis was carried out with the CONTIN deconvolution algorithm [30] at the DichroWeb server [31] using protein reference set 4 [32].

### Size-exclusion chromatography with multi-angle light scattering

Size-exclusion chromatography (SEC) with multi-angle light scattering (SEC-MALS) was performed at room temperature. A sample (500 µl) of purified TWA1 at a concentration of 2 mg/ml was injected on to an S200 300/10 GL analytical size-exclusion column (GE Healthcare) attached to a light scattering diode array (Dawn Heleos II, Wyatt Technology, U.K.) and a differential refractive index detector (Optilab rEX, Wyatt Technology, U.K.). The column was pre-equilibrated at room temperature with buffer containing 50 mM TRIS, 200 mM NaCl and 0.5 mM tris(2-carboxyethyl)phosphine (TCEP). Detector normalization was achieved by the use of BSA (Sigma–Aldrich).

### SDS/PAGE

To denature proteins for PAGE, samples were boiled for 10 min in SDS/PAGE sample buffer. In some experiments, samples with a final volume of 30 µl were separated on 10% (w/v) polyacrylamide gels under reducing conditions. In other experiments, samples with a final volume of 10 µl were loaded on NuSEP TRIS–glycine gels. Electrophoresis with handcast gels was performed in a GIBCO BRL Vertical Gel Electrophoresis apparatus with running buffer of 0.2 M glycine, 0.025 M TRIS and 0.1% SDS at 45 V. Electrophoresis with precast NuSEP gels was performed in a Mini Protein II gel apparatus (Bio-Rad) with TRIS–glycine running buffer at 130 V.

### Native PAGE

Samples were mixed 1:1 with native PAGE gel loading buffer (final concentrations, 87 mM TRIS, 0.2% bromophenol blue and 0.004% glycerol, containing maximum of 1 mM DTT) immediately prior to loading on to 10% native polyacrylamide gels. The resolving gel contained 375 mM TRIS, pH 8.8. The stacking gel contained 625 mM TRIS, pH 6.8. Electrophoresis was performed with native PAGE running buffer (0.19 M glycine, 25 mM TRIS, pH 8.8) at 15V.

### Western blotting

After resolution by SDS/PAGE, proteins were transferred electrophoretically to 0.22 µm pore PVDF membrane (Millipore) by semi-dry blotting (Bio-Rad), in transfer buffer consisting of 0.19 M glycine, 25 mM TRIS, pH 7.1 and 20% methanol. Membranes were blocked for 1 h in Western blot blocking buffer (2% [w/v] powdered milk in 1× TRIS buffered saline at pH 7.5 containing 0.2% Tween 20) and then incubated in a sealed polythene bag with mouse monoclonal antibody to V5 tag (1:2500; Clontech), in 12 ml of Western blot blocking buffer for 3 h at room temperature with rotation at 350 rpm. Membranes were then washed three times in 100 ml of fresh blot blocking buffer, for 5 min each wash, with rotation at 100 rpm. Membranes were then incubated in a sealed bag with goat anti-mouse IgG conjugated to alkaline phosphatase (1:5000; Life Technologies) in 10 ml fresh Western blot blocking buffer for 1 h at room temperature with rotation at 350 rpm. Membranes were washed three times as described above, rinsed twice in TRIS-buffered saline and developed in 6 ml of Western blot assay buffer (1 mM MgCl_2_ in 1% (v/v) diethanolamine containing 60 µl chloro-5-substituted adamantyl-1,2-dioxetane phosphate [CSPD; Life Technologies]) for 5 min. Membranes were covered in a polythene sleeve and exposed to ECL Hyperfilm (Amersham) that was developed in a CURIX 60 (AFGA) X-ray processor.

### Mass spectrometry

Protein bands were cut into 1 mm^2^ pieces and digested with sequencing grade trypsin (Promega) using a ProGest automated digestion unit (Digilab Ltd.). The resulting peptides were analysed by MS. Mass spectra were recorded in positive ion reflector mode on an Applied Biosystems 4700 MALDI mass spectrometer. For MS/MS analysis, the top five most intense, non-tryptic precursors were selected for fragmentation by collision-induced dissociation. Neither baseline subtraction nor smoothing was applied to recorded spectra. MS and MS/MS data were analysed using GPS Explorer 3.5 (Applied Biosystems). MS peaks were selected between 800 and 4000 Da and filtered with a minimum signal-to-noise ratio of 15 and to exclude masses derived from trypsin autolysis. MS/MS peaks were filtered to exclude peaks with a signal-to-noise ratio less than 10 over a mass range of 50 Da to 20 Da below the precursor mass.

Data were analysed using the MASCOT algorithm (Matrix Science) and searched against the NCBI Human protein database. A maximum number of missed cleavages of 1 and a charge state of +1 were assumed for precursor ions. Peptide precursor mass tolerance was set at 100 ppm, and MS/MS tolerance was set at 0.25 Da. Search criteria included carbamidomethylation of cysteine as a fixed modification and oxidation of methionine as a variable modification. A MASCOT score greater than 64 (the default MASCOT threshold for such searches), corresponds to a statistically significant (*P*<0.05), confident identification. TWA1 alone was recovered from the sample with a MASCOT score >65 (See Supplementary Data).

## Results and discussion

### Bacterial expression of TWA1.V5His6

The bacterial expression plasmid used, pET151/D-TOPO, supplies an N-terminal V5 and His6 tag with a following TEV cleavage site. Pilot experiments were carried out with 5 ml *E. coli* cultures (as described in the Materials and methods section) to determine conditions suitable for IPTG-induced expression of TWA1.V5His6. Cell lysates were prepared at several timepoints, either with a commercial extraction buffer (Xtractor) or by sonication of bacterial suspensions. The supernatant and pellet fractions were analysed by SDS/PAGE after reduction by DTT. A strong band of TWA1.V5His6 was present in the pellet of induced, Xtractor-lysed cells after 3 h of induction at 37°C. This protein resolved with an apparent molecular mass of 33 kDa, consistent with the predicted molecular mass of TWA1 plus the V5 and 6-His tags (Figure 1B, lane 5). However, the supernatant fraction contained little TWA1.V5His6 (Figure 1B, lane 4). TWA1.V5His6 was also detected in the pellets of sonicated cells after 3h, 5h or 16h of IPTG-induction at 37°C (Figure 1B) and small amounts of TWA1.V5His6 were detected in supernatants from sonicated cells at timepoints beyond 3 h (Figure 1B). Combining growth of *E. coli* at 20°C during the induction period with lysis by sonication increased the amount of TWA1.V5His6 in the supernatant fraction, after either 5 h or overnight induction times (Figure 1). As a compromise between optimizing protein folding conditions (favoured by lower temperature and tapered growth period) and maximizing protein yield (favoured by the most effective lysis method and maximal growth period), 5 h was chosen as the IPTG induction period, 20°C was chosen as the growth temperature during IPTG induction and cell lysis by sonication was chosen as the extraction method for larger-scale purifications of TWA1.V5His6.

### Purification of TWA1.V5His6

**Figure 2:**
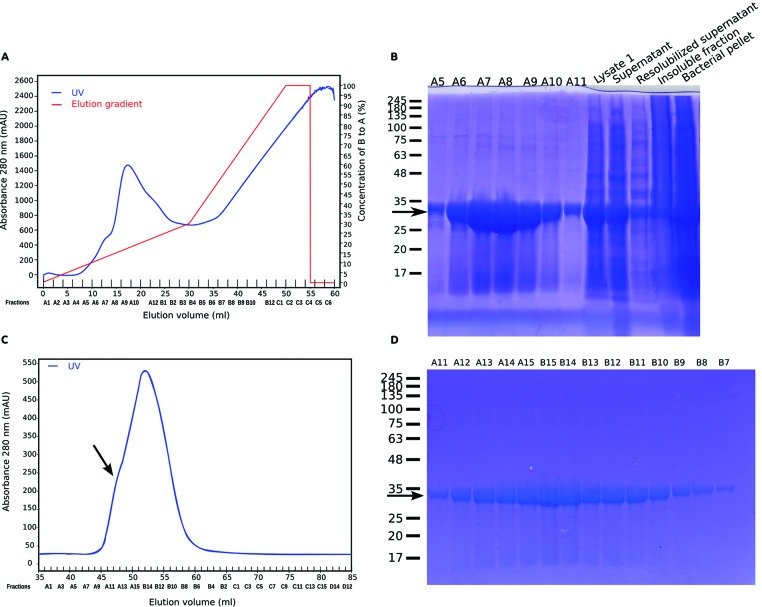
Purification of bacterially expressed TWA1.V5His6 **(A)** UV trace from metal affinity chromatography of lysates of sonicated *E. coli* containing TWA1.V5His6. (**B)** SDS/PAGE analysis of equal volumes (30 μl) of fractions A5–A11 across the main protein peak from the metal affinity chromatography along with samples of the total bacterial pellet (“pellet”), initial lysate (“Lysate”) and supernatant after centrifugation (“supernatant). For comparison, supernatant “resolubilized supernatant”) and pellet (“insoluble pellet”) after 8 M urea extraction are shown. Arrow indicates TWA1.V5His6. **(C)** UV trace from SEC of the pooled peak TWA1.V5His6 fractions from the metal affinity chromatography step. Arrow indicates the shoulder peak. (**D)** SDS/PAGE analysis of equal volumes of fractions across the protein peak from the SEC. Arrow indicates TWA1.V5His6. In (**B**) and (**D**), molecular mass markers are given in kDa.

With use of larger scale *E. coli* liquid cultures, TWA1.V5His6 was prepared and purified from the cell lysate supernatant of sonicated *E. coli* by nickel affinity chromatography and elution under a 25 mM to 1 M gradient of imidazole. The major peak of protein eluted between 0.186 M and 0.206 M imidazole (Figure 2A). When fractions around the protein peak, along with samples from earlier steps of the protocol, were analysed on TRIS–glycine gels under reducing conditions, a strong band of approximately 33 kDa was apparent with peak protein between fractions A6 and A10 (arrowed in Figure 2B). This protein was identified as TWA1 by MALDI MS analysis of the band from fraction A9 (as in Figure 2A; see also Supplementary Data).

The TRIS–glycine gel analysis also indicated that the fractions that contained the most TWA1.V5His6 also included small amounts of higher molecular mass bands (Figure 2B). Therefore, the peak TWA1.V5His6 fractions from the metal affinity chromatography step were pooled together and fractionated by SEC. The analysis yielded a single major protein peak (Figure 2C). When fractions across the peak were analysed by TRIS–glycine gel electrophoresis under reducing conditions, all fractions contained a major, monodisperse protein band that resolved with a relative molecular mass of approximately 33 kDa and corresponded to TWA1.V5His6 (arrowed in Figure 2D). Yields were approximately 20 mg/l.

**Figure 3:**
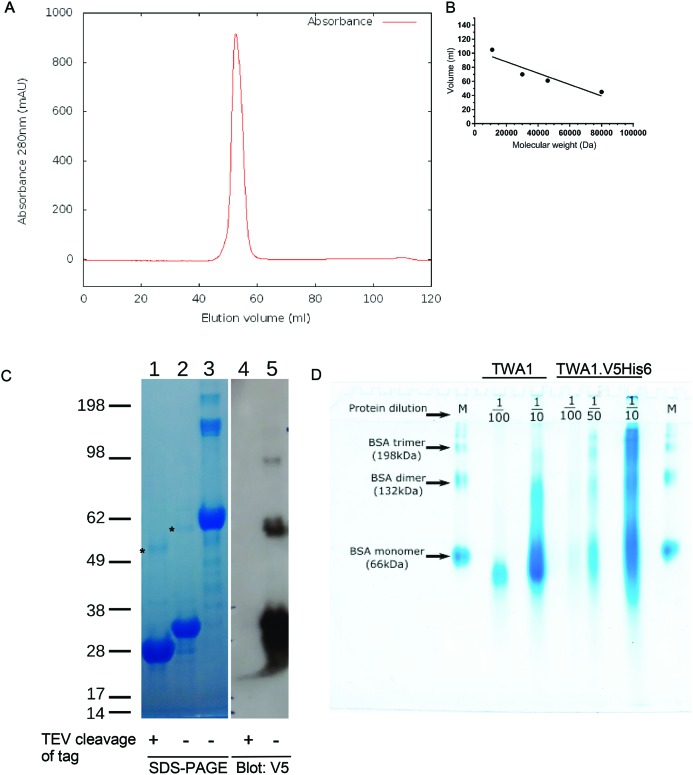
Evidence for thiol-independent dimerization of TWA1 **(A)** UV trace from SEC of TWA1 purified in the presence of TCEP and removal of the V5His6 tag by TEV protease. (**B)** Calibration curve of elution volumes of molecular mass standards from SEC. **(C)** PAGE analysis by reducing conditions on a TRIS–glycine gel of purified, recombinant TWA1 before and after removal of the V5His6 tag by TEV protease. Lanes 1–3: Coomassie blue stained gel. Lanes 4 and 5: immunoblot of replicate lanes on the same gel with V5 antibody. Lanes 1 and 4, TWA1 after tag removal; lanes 2 and 5, TWA1.V5His6; lane 3, BSA. Six micrograms of TWA1 were loaded per lane. Molecular mass markers are given in kDa. (**D)** Native PAGE separation of purified TWA1 samples, without or with removal of His6 tag. The TWA1 preparations were diluted as indicated to achieve different protein loads/lane. BSA was used as a size marker (M). Electrophoresis was carried out on 8% polyacrylamide gels and proteins visualized by Coomassie blue staining.

The absorbance traces from the SEC also indicated a shoulder peak (arrowed in Figure 2C). To investigate the possibility that TWA1.V5His6 tag might undergo artifactual aggregation, the purification strategy was modified in two ways. Firstly, purifications of TWA1 were carried out in which pooled TWA1 containing fractions from the nickel affinity chromatography step were incubated with TEV to cleave off the portion of the polypeptide containing the N-terminal V5 and His6 tags. The protein was then re-run through the nickel affinity column to retain the V5His6 tag fragment. Tag-free TWA1 was recovered in the flow through. Secondly, TWA1 has a single cysteine residue (Cys^139^) and it is possible that intermolecular thiol–thiol interactions of Cys^139^ could be responsible for dimerization of TWA1. Therefore, the reducing agent TCEP was included in all buffers to maintain thiol groups in a reduced state throughout all purification steps. TCEP was chosen over DTT, a widely used and less costly reducing agent, because TCEP is compatible with most metal affinity matrices and is a more stable reducing agent that can be used at lower concentrations [33]. Analysis of the final purified material by SEC demonstrated a single major protein peak with no shoulder (Figure 3A). In relation to the elution volumes of a set of molecular mass standards (Figure 3B), TWA1 eluted with an apparent molecular mass of around 60 kDa. Effective removal of the tags by TEV was confirmed by examination of TWA1.V5His6 compared with TWA1 after TEV cleavage and V5His6-tag fragment removal by reducing SDS/PAGE and immunoblotting. BSA was also run on the gel for additional corroboration of apparent molecular masses (Figure 3C, lane 3). Uncleaved TWA1.V5His6 was detected on the SDS/PAGE gel as a major 33 kDa band and a minor band of approximately 60 kDa (Figure 3C, lane 2, upper band marked with asterisk). These identifications were confirmed by detection of the same bands by immunoblotting with antibody to the V5 tag. An additional V5-positive minor band of approximately 98 kDa was also detected on the blot (Figure 3C, lane 5). After cleavage of the tags, the major TWA1 band on the SDS/PAGE gel had an apparent molecular mass of 28 kDa and the molecular mass of the minor approximately 60 kDa band was correspondingly reduced (Figure 3C, lane 1, upper band marked with asterisk). Neither species was detected on immunoblot by the antibody to the V5 tag, confirming the specificity of the antibody and the effectiveness of the TEV reaction (Figure 3C, lane 4). Interestingly, the persistence of the approximately 60 kDa species after removal of the V5His6 tag and purification in the presence of TCEP implicated the possibility of an unusually stable and thiol-independent mechanism of dimerization. Additional evidence for this possibility was obtained from analysis of TWA1 on native PAGE gels under non-reducing conditions. To assist visualization and interpretation, several different protein loads were analysed for each sample. TWA1.V5His6 ran at a range of molecular masses, suggestive that the tagged protein did have a tendency to aggregate. In contrast, the predominant species of tag-free TWA migrated close to the reference BSA monomer, with an apparent molecular mass of approximately 60 kDa. No monomeric (33 kDa) TWA1 was detected on these gels (Figure 3D). These results provided further indication that TWA1 forms stable dimers.

### Biochemical analysis of recombinant TWA1

**Figure 4:**
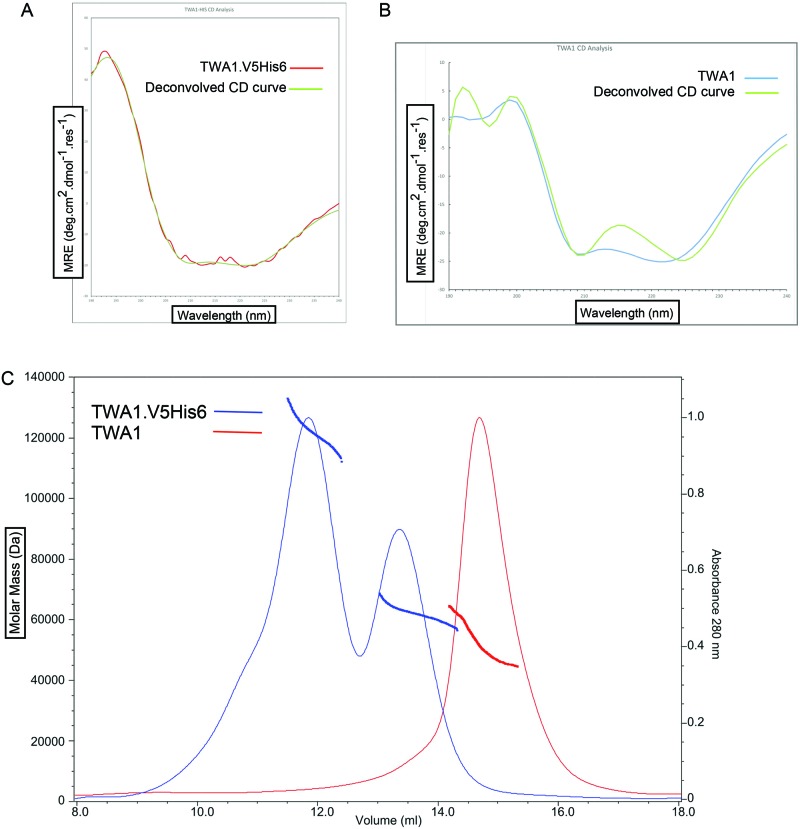
Circular dichroism and SEC-MALS analysis of TWA1 **(A)** CD spectrum of TWA1.V5His6 (from 8 scans). **(B)** CD spectrum of tag-free TWA1 (from 32 scans). Both **(A)** and **(B)** also include a superimposed, deconvolved CD curve using Contin-4 and -7 reference sets (dichroweb.cryst.bbk.ac.uk). **(C)** SEC-MALS chromatograms of the elution of TWA1.V5His6 or tag-free TWA1 from a S200 10/300 GL analytical column. SEC MALS analysis of TWA1 before, (blue) and after, (red), cleavage of the V5His6 tag.

Next, the biophysical properties of TWA1.V5His6 and tag-free TWA1 were investigated. TWA1 is predicted to be mostly α-helical [17]. The secondary structure of recombinant TWA1.V5His6 or TWA1 was assessed by CD spectroscopy. The CD spectra obtained with TWA1.V5His6 showed minima at 208 nm and 222 nm, indicative of a predominantly α-helical structure and also had a good fit with the predicted CD trace of an entirely α-helical protein (Figure 4A). Similarly, the CD spectra obtained with tag-free TWA1 had all the characteristics of an α-helical protein (Figure 4B).

To investigate the oligomeric state of recombinant TWA1.V5His6 or TWA1 in solution, SEC-MALS analysis was carried out. For TWA1.V5His6, the largest peak, representing the earliest eluted protein, corresponded to a mass of 122 kDa, as averaged across the peak. A smaller peak, representing later eluted protein, corresponded to an average mass of approximately 63 kDa (Figure 4C, blue line trace). These masses are consistent with tetrameric and dimeric forms of TWA1.V5His6 respectively. For tag-free TWA1, the single peak of later eluted proteins corresponded to an average molecular mass of 52 kDa (Figure 4C, red line trace). This mass is most consistent with dimeric TWA1. The upward slope in the molecular mass range raises the possibility that TWA1 is in equilibrium between different oligomeric states.

Taken together, these data indicate that purified TWA1 undergoes constitutive dimerization in solution. Dimer assembly does not depend on thiol–thiol interactions. In view that the LisH domain has known roles in the dimerization of other proteins such as Lis1 [21], we suspect that the LisH domain has a role in TWA1 dimerization. Indeed, residues that are important in the LisH domain of Lis1, DCAF and FOP1 for dimerization [21–23,28] are conserved strongly in TWA1 orthologues [17]. A recent crystal structure of the LisH and CTLH containing transcriptional repressor, TPL2, also indicates that the LisH domain is involved in dimerization [34]. We suspect that the LisH domain, possibly in cooperation with the CTLH region that also has α-helical characteristics [17,20], may mediate dimerization of TWA1.

In conclusion, we report a method for bacterial expression and purification of TWA1 in which TWA1 can be prepared and rendered tag-free in quantities suitable for biophysical and biochemical studies. The initial biochemical analyses reported here demonstrate constitutive, thiol-independent dimerization of recombinant TWA1. This finding has implications for further analysis of the central role of TWA1 as a highly conserved component in the multi-protein E3 ubiquitin ligases of the yeast GID complex, the metazoan muskelin/RanBP9/CTLH complex and related complexes of other eukaryotes. It will be of future interest to identify the dimerization interface and generate mutants impaired for dimerization through further biophysical and structural studies of recombinant TWA1.

## Supporting data

The data set(s) supporting the results of this article in the Supplementary Data.

## Abbreviations

CRA: C-terminal CT11-RanBP9
CTLH: C-terminal to LisH
DCAF: DDB1 and CUL4 associated factor
FOP: FGFR1 Oncogene Partner
GID: glucose-induce-degradation deficient
Lis1: lissencephaly-1
LisH: lissencephaly-1 homology
MAEA: macrophage-erythrocyte attachment protein
RING: Really Interesting New Gene domain
SEC: size-exclusion chromatography
SEC-MALS: size-exclusion chromatography with multi-angle light scattering
TCEP: tris(2-carboxyethyl)phosphine
TEV: Tobacco Etch Virus nuclear inclusion α endopeptidase protease
TWA1: two hybrid associated protein 1 (aka chromosome 20 open reading frame 11)
WDR: WD repeat domain containing
